# Influence of Perioperative Protein Supplementation on the Length of Hospital Stay and Complication Rates in Patients Undergoing Elective Abdominal Surgery

**DOI:** 10.7759/cureus.110814

**Published:** 2026-06-14

**Authors:** Nayab Ahsan, Rana Basit Ali Rajput, Sana Akhtar Ansari, Muhammad Yaqoob Hassan, Muhammad Arslan Riaz, Hafiz Muhammad Shoaib, Khushnood Ali

**Affiliations:** 1 Internal Medicine, Quaid-e-Azam Medical College, Bahawalpur, PAK; 2 Cardiothoracic Surgery, Wythenshawe Hospital, Manchester University NHS Foundation Trust, Manchester, GBR; 3 General Surgery, Faisalabad Medical University, Faisalabad, PAK; 4 Hepatobiliary and Liver Transplant, Shaikh Zayed Hospital, Lahore, PAK; 5 Cardiology, Punjab Institute of Cardiology, Lahore, PAK; 6 Pediatric Surgery, Children Hospital Lahore, Lahore, PAK

**Keywords:** abdominal surgery, nutritional status, patient, postoperative complications, ­wound healing

## Abstract

Background: Nutritional status plays a crucial role in postoperative recovery, yet perioperative protein intake is often overlooked in routine surgical care.

Objective: The objective of this study is to determine the influence of perioperative protein supplementation on the length of hospital stay and postoperative complication rates in patients undergoing elective abdominal surgery.

Methods: This observational cohort study was conducted at Shaikh Zayed Hospital, Lahore, from June 2025 to December 2025. A total of 265 adult patients undergoing elective abdominal surgery were included and categorized into protein supplementation and non-supplementation groups. Patients in the supplementation group received a high-protein oral nutritional formula targeting a total protein intake of approximately 1.2-1.5 g/kg/day, initiated 5-7 days preoperatively, where feasible, and continued for at least five postoperative days or until discharge. Baseline demographic, clinical, and surgical characteristics were generally comparable between groups. Postoperative complications, wound healing status, and length of hospital stay were compared.

Results: Patients receiving protein supplementation had a significantly shorter hospital stay compared with those without supplementation (4.8 ± 1.9 vs. 6.2 ± 2.4 days, p = 0.001). Overall postoperative complications were lower in the supplementation group than in the non-supplementation group (18.2% vs. 33.8%, p = 0.004). Infectious complications were also reduced (9.8% vs. 20.3%, p = 0.018), including surgical site infections (5.3% vs. 12.0%, p = 0.047). Optimal wound healing by postoperative day three was more frequent among supplemented patients (84.9% vs. 63.1%, p < 0.001).

Conclusion: Perioperative protein supplementation was associated with shorter hospital stays, fewer postoperative complications, and improved early wound healing among patients undergoing elective abdominal surgery. Further randomized studies are recommended to confirm these findings and establish causality.

## Introduction

Elective abdominal surgery is commonly performed worldwide and remains associated with considerable postoperative morbidity despite advances in anesthesia, surgical technique, perioperative monitoring, and enhanced recovery pathways [1]. Postoperative recovery is influenced by several patient-related and procedure-related factors, among which nutritional status has gained increasing clinical importance. Inadequate nutritional reserves before surgery and reduced oral intake after surgery may impair immune function, delay wound healing, prolong gastrointestinal recovery, and increase the risk of postoperative complications [2]. The physiology of stress reaction to surgery might also change glucose metabolism and protein catabolism, hence leading to malnutrition development [3]. Malnutrition has been linked to a high possibility of infections, delayed wound healing, lengthened recovery, higher healthcare expenses, and mortality [4]. Malnutrition among surgical patients should be avoided by evaluating the nutritional condition and offering the necessary support. European Society of Clinical Nutrition and Metabolism (ESPEN) advises that the nutrition status of the patients should be assessed and risks associated with nutrition should be considered during the first 24 hours of admission at the hospital [5]. Research has revealed that patients with malnutrition have a higher length of stay and slow gastrointestinal tract recovery relative to well-nourished patients [6]. A study conducted by Manou-Stathopoulou et al. also discovered that a third of patients undergoing surgery were at risk of malnutrition [7]. All the recovery processes are based on protein: muscle repair, production of collagen, production of immunoglobulins, and functional reserve. However, this is the unpleasant reality that a large percentage of patients who come to us with an elective abdominal surgery are already malnourished or even protein-depleted despite looking perfectly healthy on the outside. The presence of such factors as chronic inflammation, gastrointestinal disease, malignancy, decreased oral intake either because of pain or fear of symptoms, and increased waiting times play their silent role in contributing to malnutrition [8]. This means that they have depleted their physiological resilience at the preoperative stage, even before the surgery is started. The use of Enhanced Recovery After Surgery (ERAS) protocols has already made the necessity of carbohydrate loading in the preoperative phase and early postoperative feeding lawful, yet protein supplements are inconsistently utilized [9]. It is almost ironical we drive the patients into early mobilization and physiological recovery, and the very material needed to restore a muscle and immune defense is occasionally neglected [10]. There is growing research that postoperative supplementation with specific proteins can help decrease postoperative infectious complications, maintain lean muscle mass, and enhance functional outcomes [11]. Nevertheless, the data is distributed all over the patient populations, protein regimens, and intervals of supplementation. The length of hospital stay is among the most delicate measures of surgical recovery [12].

Objective

The objective of this study is to determine the influence of perioperative protein supplementation on the length of hospital stay and postoperative complication rates in patients undergoing elective abdominal surgery.

## Materials and methods

This observational cohort study was conducted at Shaikh Zayed Hospital, Lahore, from June 2025 to December 2025. A total of 265 adult patients scheduled for elective abdominal surgery were included in the study. Participants were recruited using non-probability consecutive sampling, where the participants had to fit the eligibility criteria. The inclusion criteria were that the participants must be 18 years and above with an elective abdominal surgery scheduled and must be able to absorb enteral or oral nutrition. Patients who had undergone regular preoperative nutritional counseling and had signed informed consent were eligible as well. These were criteria that only reliable surgical subjects who could take dietary supplements were tested. Exclusion criteria were used to remove confounding medical conditions that may have an independent impact on protein metabolism or surgical recovery. Patients undergoing emergency abdominal surgery, those with severe hepatic or renal dysfunction, active preoperative infection, pregnancy, prolonged postoperative intensive care unit stay, incomplete records, or missing postoperative outcome data were excluded. Patients unable to tolerate oral or enteral nutrition within 24 hours after surgery were also excluded because the study aimed to evaluate early perioperative protein supplementation delivered through oral or enteral routes. This criterion was applied to avoid exposure misclassification; however, it may have excluded higher-risk patients with complex procedures, delayed feeding, postoperative ileus, or early complications.

Data collection

Data were collected using a structured proforma that captured preoperative demographics, nutritional indicators including BMI and serum albumin, comorbidities, and details of the surgical procedure. Information regarding protein supplementation, such as timing, dosage, and adherence, was recorded carefully. Patients were not randomly assigned to study groups. Group allocation was based on routine clinical practice, including nutritional assessment, physician recommendation, patient acceptance, availability of supplementation, and feasibility of oral or enteral intake. This real-world allocation approach was used to evaluate outcomes under routine perioperative care conditions. However, because non-random allocation may introduce selection bias, baseline characteristics were compared between groups, and multivariable analyses were planned to adjust for potential confounding variables. Postoperative monitoring included documentation of wound status, complications, infections, ileus, and any deviations from expected recovery. The length of hospital stay was calculated using standardized discharge criteria. The primary outcomes assessed were the duration of hospital stay and the overall postoperative complication rate. Secondary outcomes included wound healing characteristics, the occurrence of infectious versus non-infectious complications, and the need for re-interventions or hospital readmissions within 30 days. Protein supplementation data were collected prospectively from perioperative nutrition charts, physician orders, and nursing administration records and verified against patient medication administration logs. Protein supplementation was provided according to a standardized institutional perioperative nutrition protocol. Patients in the supplementation group received a commercially available high-protein oral nutritional formula in addition to the routine hospital diet. The target total protein intake was approximately 1.2-1.5 g/kg/day. Supplementation was initiated 5-7 days before surgery, where feasible, and continued for at least five postoperative days or until discharge. Each serving provided approximately 20-25 grams of protein and was administered twice daily. Adherence was monitored through nutrition charts, nursing administration records, and patient intake documentation. Patients who received less than 80% of the prescribed supplementation were identified during data review. A structured proforma was used to record the type of protein formulation, timing of initiation (preoperative, postoperative, or both), daily dosage, route of administration (oral or enteral), and duration of supplementation. To ensure standardization, supplementation followed a predefined institutional protocol aligned with perioperative nutrition guidelines. Patients in the supplementation group received a commercially available high-protein oral formula providing approximately 1.2-1.5 g/kg/day of total protein intake, initiated 5-7 days preoperatively where feasible and continued for at least five postoperative days or until discharge. Adherence was monitored daily through intake documentation, and patients who failed to receive at least 80% of the prescribed protein dose were flagged during data abstraction. This standardized approach minimized variability in exposure and allowed consistent comparison between supplementation and non-supplementation groups. Protein supplementation was defined according to a standardized perioperative nutritional protocol implemented at the study center. Patients in the supplementation group received a commercially available high-protein oral nutritional formula in addition to the routine hospital diet. The target protein intake was 1.2-1.5 g/kg/day of total protein, consistent with perioperative nutrition guidelines for surgical patients. The supplement itself provided approximately 20-25 grams of protein per serving, administered twice daily, beginning 5-7 days before surgery when feasible and continued for at least five postoperative days or until discharge.

Data analysis

All data were analyzed using IBM SPSS Statistics for Windows, Version 25 (Released 2017; IBM Corp., Armonk, New York, United States). Continuous variables such as age, BMI, and hospital stay were summarized using mean and standard deviation, while categorical variables were expressed as frequencies and percentages. Between-group comparisons were performed using independent t-tests for quantitative outcomes and chi-square tests for categorical variables. To account for potential confounding, multivariable regression analyses were performed. Multivariable logistic regression was used for binary outcomes, including overall postoperative complications, infectious complications, surgical site infection, delayed wound healing, readmission, and re-intervention. A p-value of 0.05 or less was considered statistically significant.

## Results

Data were collected from 265 patients; the mean age of patients in the protein supplementation group was 48.1±13.4 years, which is almost the same as that of 47.7±13.8 years in the non-supplementation group. Gender distribution was similar between the groups, with 70 male patients and 62 female patients in the supplementation group compared with 68 male patients and 65 female patients in the non-supplementation group. There was also a match in the values of BMI, 24.9±3.8 kg/m^2^ vs 25.1± 4.1 kg/m^2^. There were 34 patients with diabetes (25.8%) in the supplementation group and 38 patients with diabetes (28.6) in the non-supplementation group. Hypertension was found in 41 patients (31.1%) and 39 patients (29.3%). The non-supplementation group had slightly more smokers at 27 patients (20.3 percent) as compared to the 22 patients (16.7 percent). Supplemented and control patients had higher levels of serum albumin of 3.9±0.4g/dl and 3.6±0.5g/dl, respectively. On the type of surgery, 78 patients (59.1) in the supplementation group and 75 patients (56.4) in the non-supplementation group had laparoscopic surgery, whereas 54 patients (40.9) and 58 patients (43.6) had an open surgery, respectively. Surgery time was not different (128±34 vs 131±37 minutes), and intraoperative blood loss was 185±72 vs 198±84 mL. With American Society of Anesthesiologists (ASA) class distribution (I/II/III), there was close correspondence at 48/62/22 vs 45/68/20 (Table 1).

**Table 1:** Baseline Characteristics of Patients (N = 265) ASA: American Society of Anesthesiologists

Variable	Protein Supplementation (n = 132)	No Supplementation (n = 133)
Age (years), mean ± SD	48.1 ± 13.4	47.7 ± 13.8
Gender (Male/Female)	70/62	68/65
BMI (kg/m²), mean ± SD	24.9 ± 3.8	25.1 ± 4.1
Diabetes, n (%)	34 (25.8)	38 (28.6)
Hypertension, n (%)	41 (31.1)	39 (29.3)
Smoking, n (%)	22 (16.7)	27 (20.3)
Serum Albumin (g/dL), mean ± SD	3.9 ± 0.4	3.6 ± 0.5
Type of Surgery: Laparoscopic, n (%)	78 (59.1)	75 (56.4)
Type of Surgery: Open, n (%)	54 (40.9)	58 (43.6)
Duration of Surgery (minutes), mean ± SD	128 ± 34	131 ± 37
Intraoperative Blood Loss (mL), mean ± SD	185 ± 72	198 ± 84
ASA Class I/II/III	48/62/22	45/68/20

Total complications occurred in 24 patients (18.2%) compared with 45 patients (33.8%) in the non-supplementation group. Infectious complications were reported in 13 patients (9.8%) with supplementation and in 27 patients (20.3%) without it. Surgical site infections occurred in seven patients (5.3%) in the supplementation group and 16 patients (12.0%) in the non-supplementation group. Non-infectious complications occurred in 11 patients (8.3%) with supplementation and 18 patients (13.5%) without supplementation. Postoperative ileus was documented in six patients (4.5%) and 11 patients (8.3%), while anastomotic leaks occurred in one patient (0.8%) compared with four patients (3.0%) (Table 2).

**Table 2:** Postoperative Complications in Patients

Complication Type	Protein Supplementation (n = 132)	No Supplementation (n = 133)	p-value
Total complications, n (%)	24 (18.2)	45 (33.8)	0.004
Infectious complications, n (%)	13 (9.8)	27 (20.3)	0.018
Non-infectious complications, n (%)	11 (8.3)	18 (13.5)	0.17
Surgical site infection, n (%)	7 (5.3)	16 (12.0)	0.047
Post-op ileus, n (%)	6 (4.5)	11 (8.3)	0.21
Anastomotic leak, n (%)	1 (0.8)	4 (3.0)	0.18

The mean hospital stay was shorter among supplemented patients at 4.8 ± 1.9 days, compared with 6.2 ± 2.4 days in those without supplementation. A total of 104 patients (78.8%) in the supplementation group were discharged within five days, while only 71 patients (53.4%) in the non-supplementation group met the same benchmark. Longer hospital stays beyond five days occurred in 28 patients (21.2%) with supplementation and in 62 patients (46.6%) without supplementation. Wound healing outcomes also favored the supplementation group: optimal healing by day three was seen in 112 patients (84.9%) compared with 84 patients (63.1%) in the non-supplementation group (Table 3).

**Table 3:** Length of Hospital Stay

Variable	Protein Supplementation (n = 132)	No Supplementation (n = 133)	p-value
Hospital stay (days), mean ± SD	4.8 ± 1.9	6.2 ± 2.4	0.001
Discharged ≤5 days, n (%)	104 (78.8)	71 (53.4)	<0.001
Discharged >5 days, n (%)	28 (21.2)	62 (46.6)	<0.001
Optimal healing by day 3, n (%)	112 (84.9)	84 (63.1)	<0.001
Delayed healing, n (%)	20 (15.1)	49 (36.9)	<0.001
Need for wound care intervention, n (%)	8 (6.1)	22 (16.5)	0.009

Readmission within 30 days occurred in six patients (4.5%) in the supplementation group compared with 13 patients (9.8%) in the non-supplementation group. Re-intervention was also numerically lower among supplemented patients, with four patients (3.0%) versus 10 patients (7.5%) requiring further procedures. Emergency room visits without admission occurred in nine patients (6.8%) with supplementation and 14 patients (10.5%) without it (Table 4).

**Table 4:** Readmission and Re-intervention Within 30 Days

Variable	Protein Supplementation (n = 132)	No Supplementation (n = 133)	p-value
Readmission, n (%)	6 (4.5)	13 (9.8)	0.08
Re-intervention, n (%)	4 (3.0)	10 (7.5)	0.11
ER visit without admission, n (%)	9 (6.8)	14 (10.5)	0.28

## Discussion

This observational cohort study evaluated the association between perioperative protein supplementation and postoperative outcomes among patients undergoing elective abdominal surgery. The findings showed that patients who received structured protein supplementation had shorter hospital stays, lower overall complication rates, fewer infectious complications, and better early wound condition compared with patients who received standard nutritional care. However, because the study was non-randomized, these findings should be interpreted as associations rather than proof of a direct causal effect. The most notable finding was the decrease in the length of stay. The average stay of patients in the supplementation group was 4.8 days, as compared to the non-supplementation patients with an average of 6.2 days, which highlights the importance of protein in promoting physiological recovery. This is biologically plausible considering that surgical stress causes a hypermetabolic, catabolic state [13]. Sufficient protein consumption aids in collagen synthesis, immunity, and muscle maintenance, all of which have a direct impact in terms of mobilization, wound strength, and discharge preparedness. Similar increases in postoperative recovery time have been reported in previous studies when patients are given specific perioperative nutrition, which indicates that protein supplementation might be in line with other more general enhanced recovery theories. There was also a significant difference in the complication rates in the two groups [14]. The rate of overall complications was almost two-thirds less in the supplementation group compared to the non-supplementation group. There was a reduction in infectious complications such as surgical site infections and postoperative pneumonia. Protein has a prime position in immune capability, and a lack thereof has been shown to negatively affect T-cell response, antibody production, and barrier integrity. The reduced rate of complications herein attests to the idea that the outcomes of surgery are closely correlated with metabolic resilience, which can be enhanced with the help of perioperative protein consumption. Past studies also show that malnutrition is a risk factor for postoperative infections, and a positive change in protein status is associated with a lessening of such occurrences [15].

The results of wound healing also highlighted the advantages of protein supplementation. Maximal wound healing in the early stage was much more prevalent in supplemented patients, indicative of increased cross-linking, fibroblast activity, and control of inflammation. The non-supplementation group had a significant number of delayed healings. This is in line with prior studies that have revealed protein deficiency to impair tensile strength, extend inflammatory response, and heighten predisposition to breakdown of wounds. Rapid wound healing is useful indirectly in shortening the length of stay as well as lowering the risk of subsequent complications. The readmission and re-intervention rates were found to be lower in the supplementation group; however, the difference was not found to be significant [16]. This can be associated with the general low rate of events, which implies that the sample size would have to be larger to identify more acute differences in these secondary outcomes. However, the decreasing trend remains consistent with the general trend of the enhanced recovery observed in other variables [17]. There are a few limitations that should be recognized in this study. The observational cohort design limits causal interpretation, as patients were not randomly assigned and group allocation depended on clinical assessment, physician recommendation, patient acceptance, availability of supplementation, and feasibility of early oral or enteral intake, which may have introduced selection bias. Baseline nutritional status was also not fully balanced, as serum albumin was higher in the supplementation group, suggesting better preoperative nutritional reserves; although adjusted analysis was performed, residual confounding from unmeasured factors such as dietary intake, muscle mass, inflammatory status, and socioeconomic status may remain. Elective abdominal surgery included heterogeneous procedures with different operative complexity, complication risks, and recovery patterns, and despite adjustment for surgical type, approach, operative duration, blood loss, and complexity, procedure-related confounding cannot be excluded. Excluding patients unable to initiate oral or enteral nutrition within 24 hours may also have removed higher-risk patients with complex procedures, postoperative ileus, anastomotic concerns, or early complications, limiting generalizability. Wound condition was assessed clinically on postoperative day three without a validated scale or blinded evaluation, so this outcome should be interpreted cautiously as an early wound-status indicator rather than true wound healing. 

## Conclusions

Perioperative protein supplementation was associated with shorter hospital stays, lower postoperative complication rates, and better early wound condition among patients undergoing elective abdominal surgery. However, due to the observational design and potential residual confounding, these findings should be interpreted cautiously and should not be considered evidence of a direct causal effect. Further prospective randomized studies are needed to confirm these associations and determine the optimal timing, dose, duration, and target population for perioperative protein supplementation.
